# The Healthcare Integrated Research Database (HIRD) as a Real‐World Data Source for Pharmacoepidemiologic Research

**DOI:** 10.1002/pds.70110

**Published:** 2025-02-05

**Authors:** John J. Barron, Vincent J. Willey, Brett T. Doherty, Ozgur Tunceli, Craig R. Waltz, Michael Grabner, Daniel C. Beachler, Stephan Lanes, Mark J. Cziraky

**Affiliations:** ^1^ Carelon Research Wilmington Delaware USA

**Keywords:** Carelon, claims, database, EHR, Elevance Health, Healthcare Integrated Research Database, HIRD, real‐world data, real‐world evidence, SDoH

## Abstract

**Background and Methods:**

The Healthcare Integrated Research Database (HIRD) is a large, comprehensive real‐world data (RWD) source for health‐related research. Demographic and healthcare‐related characteristics of individuals are sourced from routinely updated RWD. The HIRD includes health insurance claims and other health‐related information for individuals enrolled in health insurance plans offered or managed by Elevance Health and has been utilized for research for almost two decades. Individuals in the HIRD reside throughout the United States. Data in the HIRD have been available since January 2006, and are updated monthly.

**Results:**

As of July 2024, the researchable population of the HIRD included over 91 million individuals with medical benefits, and over 24 million individuals were actively enrolled. The median age of individuals in the HIRD is 36 years (interquartile range [IQR]: 22, 54), and 50% of individuals in the HIRD are female. The median duration of continuous enrollment in the HIRD is 2.0 years (IQR: 0.8, 4.7). For those actively enrolled, the median duration of continuous enrollment is 3.8 years (IQR: 1.7, 8.3). Other important characteristics of the HIRD include the ability to trace data back to their source, support both deterministic and probabilistic linkage with external data sources, and link family members within health plans.

**Conclusions:**

The HIRD has been a trusted resource to generate real‐world evidence for a variety of health‐related research, including regulatory‐required safety studies, comparative effectiveness studies, and health economics and outcomes research.

**Summary**

- The Healthcare Integrated Research Database (HIRD) includes health insurance claims and other health‐related information for individuals enrolled in Elevance Health insurance plans across the United States (US).
- Data in the HIRD have been available since January 2006, and are updated monthly.
- As of July 2024, the HIRD included over 91 million individuals with medical coverage, with approximately 24 million actively enrolled.
- Other important characteristics of the HIRD include the ability to trace data back to its source, support both deterministic and probabilistic linkage with external data sources, and link family members within health plans.

## 1 Introduction

Real‐world data (RWD) are increasingly used to generate real‐world evidence (RWE) to support the development and regulation of healthcare products, perform safety and effectiveness studies, and conduct health economics and outcomes research, among other uses [1, 2, 3]. In response to the expanding utility of RWE, there is increasing availability of RWD sources, including national healthcare registries, disease and exposure registries, electronic health record (EHR) systems, and healthcare claims databases [4, 5, 6, 7].

Individuals in the United States (US) can obtain health insurance from their employer, individual exchanges, or government programs (i.e., Medicare and Medicaid). For employer‐sponsored insurance, there is typically a designated open enrollment period each year, during which employees can sign up for or make changes to their health insurance plans, as well as special enrollment periods for individuals who experience qualifying life events (e.g., marriage, birth/adoption of a child). Employees receive information about the available health plans, including details on premiums, coverage options, provider networks, and out‐of‐pocket costs. Employees evaluate the plans and select one that best meets their needs and budget. Individuals who do not have access to employer‐sponsored coverage can buy insurance through individual exchanges. These exchanges have an annual open enrollment period as well as special enrollment periods for individuals who experience a qualifying life event. The exchanges provide a range of health plans to choose from, allowing individuals to compare details on premiums, coverage options, provider networks, and out‐of‐pocket costs. Medicare is generally available to individuals aged 65 and older or to those under 65 with certain disabilities or conditions. Enrollees choose between traditional Medicare, offered from the federal government (Centers for Medicare & Medicaid Services), or Medicare Advantage plans offered through private insurance companies. Those who purchase traditional Medicare can also purchase supplemental coverage from private insurance companies to help cover out‐of‐pocket costs not covered by traditional Medicare. In addition, individuals can choose prescription drug coverage (Medicare Part D) from private insurance companies approved by Medicare. Eligibility for Medicaid is primarily based on income, household size, and state‐specific criteria. If eligible, individuals may choose from various managed care plans offered by private insurers contracted with the state Medicaid program.

When a covered individual seeks medical care, healthcare providers (e.g., doctors, hospitals) deliver services and generate a claim containing service details and costs which is submitted to the insurer using standardized forms. Insurers review claims for accuracy, coverage, and medical necessity and determine the amount payable based on contractual agreements. The insurer then sends payments to providers for approved services. Insurers maintain comprehensive records of all submitted claims, whether paid or denied.

Healthcare claims databases are an attractive option to obtain RWD, as they can include the healthcare information needed to support many research activities (such as enrollment dates in the health plan, demographic characteristics, diagnoses, treatments, and costs) in large populations. This information, obtained from all eligible members, providers, and facilities, identifies time periods of health plan membership and provides a nearly comprehensive picture of a patient's interactions with the healthcare system during this time of active enrollment. Though research using claims data alone can provide significant value, it does have limitations. Claims data are not captured for services which are not paid by the health plan (i.e., individual pays cash for services), certain clinical details are not contained in claims data, and the accuracy of diagnosis and procedure coding can be variable.

There are a growing number of individual healthcare claims databases and multi‐source claims data aggregator databases available to researchers, and each database possesses attributes that impact its suitability for a specific research purpose. Foremost, a database must include the target population in sufficient numbers and the data elements necessary, with sufficient accuracy and completeness, to conduct valid and reliable research. There may be other requirements for a specific research project, such as the ability to trace the claims data to their origins to ensure satisfactory data quality, the ability to link external sources of information (e.g., medical records, National Death Index (NDI)) to supplement or validate claims data, or the ability to link mothers with their infants to conduct pregnancy‐related research.

The Healthcare Integrated Research Database (HIRD, formerly the *HealthCore* Integrated Research Database) is a data environment curated and maintained by Carelon Research that includes “closed” (reviewed and approved by the payer) healthcare claims of individuals from across the US and has been augmented with additional data to support a variety of health‐related research. The HIRD contains data from 2006 and is updated monthly. This article describes the types, elements, timeliness, and quality of the data in the HIRD for health researchers considering RWD for US populations.

## 2 Data Types in the HIRD

The foundation of the HIRD is the enrollment records and associated healthcare claims for individuals enrolled in commercial, Medicare, or Medicaid health plans offered or managed by Elevance Health (Table 1). The HIRD includes data from health plans in 33 states in the US, with individual members located throughout all 50 states. Data from individuals in these plans are generally available for research; however, some individuals or employer groups may choose not to make their data available for research. There are no restrictions on the use of data from Medicare Advantage plans (complete coverage through private health plans), supplemental Medicare coverage through the health plans, and Medicare Part D (pharmacy coverage). The HIRD does not contain traditional Medicare data. Medicaid plans require state‐by‐state approval for use in research.

**TABLE 1:** HIRD data types.

Type	Sources	Elements (selected)	Years available	Cumulative availability, *n* ^a^	Availability, % of total enrolled
Enrollment files	ELV‐affiliated health plans	Age, sex, enrollment dates, plan type, residence	2006–current	91 m	N/A
Medical claims	ELV‐affiliated health plans	Service dates and locations, provider specialty, diagnoses, procedures, paid amounts	2006–current	91 m	100%
Pharmacy claims	ELV‐affiliated health plans	Fill dates, prescriber specialty, drug name, formulation, quantity, days' supply, paid amounts	2006–current	72 m	79%^b^
Electronic Health Records^c^	EHR systems; health systems/ clinics; HIEs	Structured (e.g., vital signs; dates) and unstructured fields (e.g., physician notes)	2006–current	13 m (integrated) 55 m (available to request)	14% (integrated) 60% (available to request)
Laboratory results	Lab providers; EHR	Lipid panels, blood glucose, inflammation markers, etc.	2006–current	44 m	48%
Vital status	Enrollment data; inpatient claims; DMF from SSA; online obituaries; others^d^	Day of death (if applicable)	2006–current	91 m	100%
Oncology	Carelon Cancer Care Quality Program	Stage, pathology, line of treatment, regimen, biomarkers	2014–current	373 k	N/A^e^
Social and health equity data	Various individual‐level and area‐level data^f^	Individual‐level race and ethnicity; area‐level measures of socio‐economic status, food access, urbanicity	Individual‐level: 2006–current; area‐level: most recent data available for public use	N/A	Up to 95% (race/ethnicity); 95% (area‐level)
Vaccinations	Claims, registries	Dates, types	2006–current	91 m (claims); 55 m (registries)	100% (claims); 60% (registries)

*Note:* This table applies to individuals with commercial or managed Medicare coverage. All data types are updated monthly, except for certain area‐level social and health equity data, whose update frequency depends on public release schedules.

Abbreviations: DMF, Death Master File; EHR, electronic health records; ELV, Elevance Health; HIE, health information exchange; HIRD, Healthcare Integrated Research Database; m, million; N/A, not applicable; SSA, Social Security Administration.

^a^ Data through July 2024.

^b^ Individuals without pharmacy claims may either be covered solely for medical benefits and pay for prescription drugs out of pocket, or may have pharmacy benefits with a separate insurer and no administrative services from ELV. In many studies, the eligible research population is therefore restricted to individuals with both medical and pharmacy enrollment.

^c^ The HIRD includes EHR integrated and readily available data for ~13 m individuals; medical records can also be requested as needed from providers for ~55 m individuals (not mutually exclusive to the ~13 m with EHR).

^d^ Full list of sources: enrollment files (reason for disenrollment), inpatient claims (discharge status), Death Master File from the Social Security Administration, utilization management data, Center for Medicare and Medicaid Services (CMS) records, and online obituary information processed by third‐party vendors.

^e^ Availability depends on the type of cancer and study period.

^f^ Individual‐level race and ethnicity data are obtained from enrollment files, EHR data, member self‐assessments, and proprietary imputation algorithms. Area‐level data are taken from public sources, including the National Center for Health Statistics' urban–rural classification scheme, the American Community Survey, and the Food Access Research Atlas.

### 2.1 Enrollment Records

Enrollment records are created for individuals for all time segments during which they are enrolled in the health plan. Knowing when individuals are eligible allows researchers to distinguish the *absence of data* from the *absence of a healthcare encounter*. These records include unique identifiers for individuals, type of health plan, period of enrollment in the health plan, and demographic and geographic information (Table 1).

Individuals typically have multiple enrollment records, or lines of enrollment data called segments, for different periods of time. For administrative reasons, there is at least one segment in a calendar year for each time period enrolled, but a single segment may span multiple years. Members may have multiple segments that can overlap or be adjacent to one another owing to changes in the member's life, including multiple addresses in the same enrollment period, change of name, or change of employer who has coverage with the same health plan. Individuals who change plans or plan types within the same overarching health plan (i.e., have multiple insurance identifiers) are linked with a master identifier so they are recognized as the same individual. This includes individuals who transition to non‐traditional Medicare coverage from individual or employee‐sponsored insurance within the same health plan. Continuous enrollment episodes are created by integrating or “rolling up” records with overlapping dates into continuous enrollment segments. Overlapping and adjacent enrollment segments are joined together with a 1‐day gap allowed. Segments separated by more than 2 days are not rolled up together, resulting in multiple enrollment records with gaps in enrollment for individual patients.

An individual can be followed across the healthcare system using unique characteristics that enable deterministic and probabilistic linkage between the individual's claims data and other data sources, including those permanently integrated into the HIRD (described below), as well as additional sources that are typically linked for specific research studies, such as medical records, the NDI, and vaccine/disease registries. Appropriate privacy and security protections are implemented to ensure regulatory compliance. The ability to link mothers and their infants within the HIRD supports pregnancy‐related research. Previous research using internally developed methodology has demonstrated that approximately 75% of infants are added to their mothers' insurance plans, allowing their data to be linked to their mothers' data in the HIRD [8, 9, 10].

### 2.2 Medical Claims

Medical claims submitted by healthcare professionals and facilities for payment of services rendered include service dates, diagnoses, procedures, provider information, service locations, and service costs (Table 1). Diagnoses are recorded using International Classification of Diseases 9th and 10th Clinical Modification codes (ICD‐9‐CM and ICD‐10‐CM), with up to 12 diagnosis codes per claim. If multiple services occur on the same date with the same provider, those services are typically combined into a single claim. Procedures, including treatment administrations such as infusions, are recorded using ICD‐9‐CM procedure codes, ICD‐10 Procedure Coding System (ICD‐10‐PCS) codes, Healthcare Common Procedure Coding System (HCPCS) codes, and Current Procedural Terminology (CPT) codes. Provider information includes National Provider Identifier (NPI) and tax identification numbers, practice name, and practice address. Service locations are classified into one of four settings: inpatient, stand‐alone emergency department, outpatient (including telehealth), and skilled nursing facility. Inpatient episodes (time period between admission and discharge) are constructed from multiple claims using a proprietary algorithm informed by service locations and service dates. Outpatient services are classified using the Restructured Berenson‐Eggers Type of Service Classification System [11]. Service costs in medical claims include the amounts paid by the health plan, the individual, and other health plans. Cost data are available for all medical claims, with a small proportion of the cost data being imputed.

### 2.3 Pharmacy Claims

Pharmacy claims for prescription treatments are submitted by outpatient pharmacies, including mail order and specialty pharmacies. Pharmacy claims include National Drug Codes (NDCs) for the treatment dispensed, quantity dispensed, days' supply, medication costs, dispensing date, prescriber information, and information about the dispensing pharmacy (Table 1). NDCs provide information about the manufacturer, medication dispensed, strength, package size, route of administration, and dosage form. NDCs are mapped to Generic Product Identifier (GPI) codes, providing further information on the medication's therapeutic category as well as medication class and sub‐class [12]. Pharmacy cost data include amounts paid by the health plan, the individual, and other health plans. For the subset of individuals who receive pharmacy benefits separately from their medical benefits, costs for pharmacy claims are imputed [13].

### 2.4 Provenance of Healthcare Claims

**FIGURE 1:**
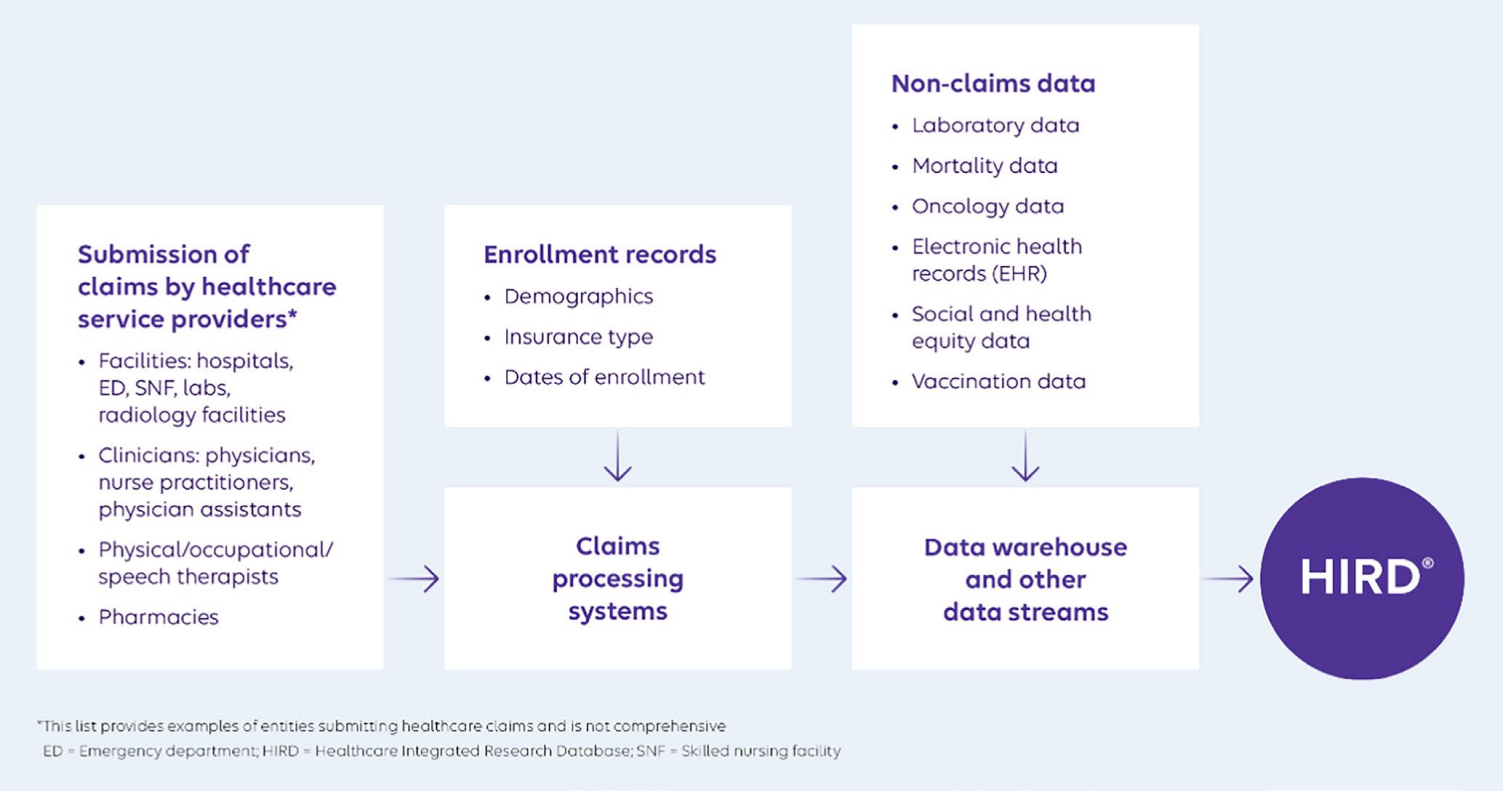
An overview of the handling of data and data types contained in the HIRD.

Healthcare claims that enter the HIRD are generated when healthcare services are provided to individuals by healthcare providers. Providers submit claims for reimbursement to the health plan, which are either accepted or rejected (and potentially resubmitted). Healthcare claims are linked to enrollment records and then processed before being stored in the data warehouse. Enrollment records and associated healthcare claims are then integrated with external data sources (Figure 1).

**FIGURE 2:**
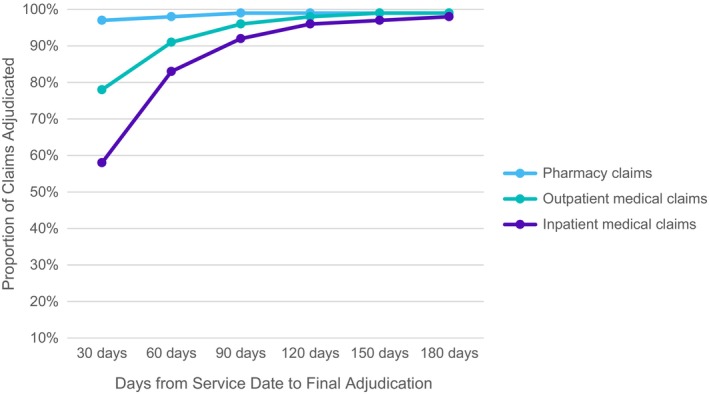
Timeliness of medical and pharmacy claim adjudication. Based upon 2024 analysis of time to final adjudication from claimservice date.

Healthcare claims data in the HIRD are updated monthly. Only paid claims are included, with over 97% of pharmacy claims paid within 30 days, more than 90% of outpatient medical claims within 60 days, and over 90% of inpatient claims within 90 days (Figure 2). As a complete healthcare claims history for a defined time period is important for many research studies, a 3‐month lag from the most recent data load is typically imposed on healthcare claims data available for research at the study level. Data in the HIRD have been available since January 2006, unless otherwise specified.

The entirety of the HIRD is updated monthly, and quality control metrics are reviewed at each update to assess the data accuracy and completeness of both old data and new incremental data (i.e., data since the previous update). Monthly updating allows the old data to be overwritten with the most current data. By reviewing the entire database each month, trends in data quality can be identified and investigated. For example, data from a large Health Maintenance Organization (HMO) plan were originally deemed incomplete due to capitation arrangements and were excluded from the HIRD due to incomplete capture of healthcare services. Through collaborative projects with the health plan in recent years, the capitated plans began submitting claims with increasing consistency, akin to non‐capitated plans. This led to a reassessment of the previous decision to exclude HMO plans. Upon verifying the enhanced completeness of claims from this large HMO, the addition of these claims to the HIRD increased the researchable population by about 5%.

Carelon Research's ability to trace data in the HIRD to their origins enables investigations of key data elements that can improve study quality. For example, in a safety study of a new migraine treatment, it was observed that the first dose recorded in the HIRD claims for many patients did not align with dosing recommendations (i.e., the first dose recorded in the HIRD claims was not the expected loading dose). The study team surveyed providers to inquire about real‐world dosing practices and learned that most providers used free medication samples for loading doses. With this knowledge, the study team was able to conduct more valid analyses and offer more meaningful interpretation of the data in the HIRD [14].

## 3 Other Data Types in the HIRD

### 3.1 Electronic Health Records

The HIRD contains structured and unstructured EHR data for a subset of individuals. EHR data are obtained from provider network systems, large health systems and clinics, and state‐level health information exchanges (Table 1). While most integrated EHR data come from outpatient primary care providers, some include records from specialists and inpatient providers. Integrated EHR data are available beginning January 2010. Additionally, the HIRD can be used as a sampling pool to identify individuals with characteristics of interest for which EHR data outside of the HIRD can be requested from providers and inpatient facilities.

Structured EHR data include anthropometrics, vital signs, behavioral risk factors, medical history, and medications prescribed. In addition to the coding systems used in medical and pharmacy claims, structured data in the EHR may also contain Systematized Nomenclature of Medicine (SNOMED) codes (diagnoses, procedures), clinical drugs normalized (RxNorm) codes (treatments), and vaccinations (CVX codes).

Unstructured EHR data include provider office visit notes. Through natural language processing, unstructured EHR data can be queried to identify clinical information and create structured fields, such as ejection fraction values for individuals with a heart failure diagnosis and heart failure classification [15].

### 3.2 Laboratory Results

Laboratory test results for outpatient laboratory services are integrated within the HIRD. Laboratory test results generated by nationwide laboratory providers or included in EHRs are defined using Logical Observation Identifiers Names and Codes (LOINC), which provide information regarding the specimen source and methods of measurement (Table 1).

Laboratory results may be reported in inconsistent formats across labs; therefore, prior to inclusion in the HIRD, they are processed and standardized to ensure logically consistent data. The HIRD includes more than 65 of the most common laboratory tests readily available for research; however, this can be expanded to include other labs of interest. The proportion of individuals with laboratory results varies by test and therapeutic area.

### 3.3 Vital Status

Vital status for individuals in the HIRD is obtained from enrollment records (reason for disenrollment), inpatient claims (discharge status), the Death Master File from the Social Security Administration, utilization management data, Center for Medicare and Medicaid Services records, and online obituary information processed by third‐party vendors. Data from these sources are combined to create a composite mortality variable for research use, indicating day of death (Table 1). Cause of death is not directly available in the HIRD but can be approximated algorithmically in many cases [16]. In a validation study among patients with advanced cancer between 2010 and 2018 (*n* = 40 679), the composite mortality variable had good agreement with the NDI (sensitivity 89%, specificity 89%, positive predictive value 93%, negative predictive value 92%) [17, 18].

### 3.4 Oncology

The HIRD includes oncology data from the Carelon Cancer Care Quality Program (CCQP). Clinical oncology data for individuals undergoing cancer treatment in outpatient settings are recorded when a healthcare provider requests preauthorization for cancer treatment. Data entered by providers into the CCQP online portal include cancer type, cancer stage, biomarkers, pathology/histology, line of treatment, planned treatment regimen, height and weight, a metric of functional status (Eastern Cooperative Oncology Group Performance Status Scale), and other clinical details (Table 1). In a validation study that compared the contents of the CCQP to medical records for breast, lung, and colorectal cancer patients, good agreement was observed for cancer type, cancer stage, histology (lung cancer only), and select cancer biomarkers [19]. CCQP data are available beginning July 2014.

### 3.5 Social and Health Equity Data

Individual‐level race and ethnicity data are obtained from enrollment files, EHR data, member self‐assessments, and proprietary imputation algorithms [20, 21]. These data are combined to create a composite race and ethnicity variable for research use (Table 1). The composite race and ethnicity variable is based on Office of Management and Budget standards (White non‐Hispanic, Native Hawaiian/Other Pacific Islander non‐Hispanic, Black/African American non‐Hispanic, Asian non‐Hispanic, American Indian/Alaska Native non‐Hispanic, Hispanic/Latino, and other race non‐Hispanic) [22]. A validation study found high agreement (Kappa = 0.82) between the composite variable and self‐reported race/ethnicity (among commercially insured Asian, Black/African American, Hispanic/Latino, and White individuals) [21].

A variety of Social Drivers of Health (SDoH) data have been integrated into the HIRD [20]. Area‐level information about urbanicity is derived from the National Center for Health Statistics' urban–rural classification scheme. The HIRD includes area‐level data from the American Community Survey, including over 50 variables at the census block group level associated with healthcare resource utilization, such as educational attainment, income, living conditions, family composition, transportation, and employment (Table 1) [23]. The HIRD also includes area‐level data from the Food Access Research Atlas, with over 140 variables at the census tract level related to food access and availability, urbanicity and rurality, and income [24]. Social and health equity data in the HIRD are available for up to 95% of individuals.

### 3.6 Vaccinations

Vaccination data from the Immunization Information System (IIS) are included in the HIRD to supplement vaccination data from healthcare claims and EHR data (Table 1). Currently, the IIS data are obtained from 16 jurisdictions in 15 states and represent approximately 60% of members in the HIRD, although the data available may differ by state [25].

## 4 Characteristics of the HIRD Population

The HIRD population used for research (“researchable population”) consists of individuals/employer groups with commercial and/or managed Medicare health insurance plans who have agreed to be included for research purposes. The specific population available for use in any given research study varies depending on population requirements for that study. Studies using claims data only can utilize the entire researchable population.

As of July 2024, the researchable population included over 91 million individuals with medical benefits: over 72 million of these individuals also have pharmacy benefits with at least 1 day of membership in the HIRD (Table 2). Of these, approximately 24 million individuals with medical benefits and approximately 17 million individuals with medical and pharmacy benefits were actively enrolled (i.e., had active health plan membership as of July 2024).

**TABLE 2:** Demographic Characteristics of the HIRD Researchable population.

	Medical benefits		Medical and pharmacy benefits	
	Enrolled at any time^a^, ^b^ (*n* = 91 753 277)	Actively enrolled on 07/31/2024^b^ (*n* = 23 934 415)	Enrolled at any time^a^, ^b^ (*n* = 72 711 460)	Actively enrolled on 07/31/2024^b^ (*n* = 16 863 080)
Age (years), median (IQR)	36 (22, 54)	40 (22, 57)	36 (22, 54)	40 (23, 56)
0–17	16 293 104 (18%)	4 241 484 (18%)	12 998 226 (18%)	3 013 925 (18%)
18–44	38 975 399 (42%)	9 123 790 (38%)	30 984 227 (43%)	6 477 569 (38%)
45–64	24 157 993 (26%)	6 946 810 (29%)	19 671 478 (27%)	5 048 125 (30%)
65–74	5 819 711 (6%)	1 943 906 (8%)	4 313 833 (6%)	1198.156 (7%)
≥ 75	3 847 300 (4%)	1 275 847 (5%)	2 399 327 (3%)	741 451 (4%)
Unknown	2 659 770 (3%)	402 578 (2%)	2 344 369 (3%)	383 854 (2%)
Sex				
Male	45 931 953 (50%)	11 877 739 (50%)	36 306 271 (50%)	8 341 032 (50%)
Female	45 783 423 (50%)	12 042 824 (50%)	36 380 229 (50%)	8 514 288 (50%)
Unknown	37 901 (0.04%)	13 852 (0.1%)	24 960 (0.03%)	7760 (0.05%)
Race/ethnicity^c^				
Individuals with available data	42 559 378 (46%)	19 058 462 (80%)	35 820 615 (49%)	13 559 120 (80%)
White, NH	27 004 414 (63%)	13 649 968 (72%)	23 117 071 (65%)	9 712 243 (72%)
Native Hawaiian or other Pacific Islander, NH	12 051 (0.03%)	2858 (0.01%)	9669 (0.03%)	2102 (0.02%)
Black and African American, NH	3 723 174 (9%)	1 503 060 (8%)	3 182 352 (9%)	1 153 867 (9%)
Asian, NH	2 832 212 (7%)	1 409 514 (7%)	2 352 205 (7%)	1 028 104 (8%)
American Indian or Alaska Native, NH	172 701 (0.4%)	94 990 (0.5%)	143 915 (0.4%)	67 509 (0.5%)
Hispanic or Latino	6 421 021 (15%)	2 115 280 (11%)	4 836 918 (14%)	1 410 459 (10%)
Other race, NH	2 393 805 (6%)	282 792 (2%)	2 178 485 (6%)	184 836 (1%)
Region				
Midwest	21 371 633 (23%)	5 692 776 (24%)	17 691 463 (24%)	4 062 778 (24%)
Northeast	14 933 776 (16%)	3 949 145 (17%)	10 637 903 (15%)	2 414 436 (14%)
South	29 751 411 (32%)	7 824 283 (33%)	24 685 349 (34%)	6 073 186 (36%)
West	24 796 250 (27%)	6 464 826 (27%)	19 005 552 (26%)	4 310 864 (26%)
Unknown	900 207 (1%)	3385 (0.01%)	691 193 (1%)	1816 (0.01%)

Abbreviations: IQR, interquartile range; NH, non‐Hispanic.

^a^ Data from January 2006 to July 2024.

^b^ Characteristics are based on the date of most recently available data.

^c^ Race and ethnicity percentages are calculated using individuals with available data as the denominator.

The median age for the researchable population is 36 years (interquartile range, IQR: 22, 54), with approximately 10% ≥ 65 years old. The researchable population is approximately half male and half female. Individuals with available race and ethnicity data (46% for the entire HIRD starting 2006; ≥ 80% for the most recent 5 years) are approximately 63% White non‐Hispanic, 15% Hispanic/Latino, 9% Black/African American non‐Hispanic, 7% Asian non‐Hispanic, and 6% other races or ethnicities. Individuals most commonly live in the South census region (32%), followed by the West (27%), Midwest (23%), and Northeast (16%).

Approximately 92% of individuals have commercial health insurance, whereas approximately 8% have managed Medicare (including Medicare Advantage, Medicare Supplement, and Medicare Part D) (Table 3). Preferred provider organizations are the most common plan type (64%), followed by HMOs (19%) and consumer‐directed health plans (17%). The median duration of continuous enrollment for the entire population is approximately 2.0 years (IQR: 0.8, 4.4). Overall, the population with medical benefits is similar to the population with medical and pharmacy benefits, and the actively enrolled population is similar to the enrolled‐at‐any‐time population (Table 3). Notably, actively enrolled patients are more likely to have race and ethnicity data (~80% for actively enrolled with medical benefits), and actively enrolled patients are typically enrolled for longer durations (median (IQR) of 3.8 (1.7, 8.3) years for actively enrolled with medical benefits).

**TABLE 3:** Health Plan Characteristics of the HIRD Researchable population.

	Medical benefits		Medical and pharmacy benefits	
	Enrolled at any time^a^, ^b^ (*n* = 91 753 277)	Actively enrolled on 07/31/2024^b^ (*n* = 23 934 415)	Enrolled at any time^a^, ^b^ (*n* = 72 711 460)	Actively enrolled on 07/31/2024^b^ (*n* = 16 863 080)
Duration of enrollment (years)^c^, median (IQR)	2.0 (0.8, 4.7)	3.8 (1.7, 8.3)	1.7 (0.7, 3.9)	3.3 (1.6, 7.3)
< 1 year	30 400 644 (33%)	2 606 845 (10.9%)	26 194 636 (36%)	2 041 220 (12.1%)
1–2 years	17 476 172 (19%)	4 382 565 (18.3%)	15 021 555 (21%)	3 306 449 (19.6%)
2–3 years	10 864 845 (12%)	3 277 054 (13.7%)	8 868 232 (12%)	2 525 352 (15.0%)
3–4 years	7 273 855 (8%)	1 956 544 (8.2%)	5 843 499 (8%)	1 564 135 (9.3%)
> 4 years	25 737 761 (28%)	11 711 407 (48.9%)	16 783 538 (23%)	7 425 924 (44.0%)
Insurance type				
Commercial	84 372 814 (92%)	21 223 554 (89%)	67 055 098 (92%)	15 210 335 (89%)
Managed Medicare	7380.463 (8%)	2 710 861 (11%)	5 656 362 (8%)	1 815 784 (11%)
Plan type				
HMO	17 623 747 (19%)	4 463 857 (19%)	16 076 244 (22%)	3 810 871 (23%)
PPO	58 316 431 (64%)	13 807 646 (58%)	42 278 668 (58%)	8 324 972 (49%)
CDHP	15 522 185 (17%)	5 508 179 (23%)	14 101 690 (19%)	4 568 590 (27%)
Other	290 914 (0.3%)	154 733 (0.7%)	254 858 (0.4%)	158 647 (1%)

Abbreviations: CDHP, consumer directed health plan; HMO, health management organization; IQR, interquartile range; PPO, preferred provider organization.

^a^ Data from January 2006 to July 2024.

^b^ Characteristics are based on the date of most recently available data.

^c^ Duration of enrollment for “Enrolled at any time” reflects the duration of the most recent continuous enrollment period (with a 32‐day allowable gap). Duration of enrollment for “Actively enrolled on 31 July 2024” reflects duration of enrollment period containing the date of the latest available data (with a 32‐day allowable gap).

## 5 Strengths and Limitations

The HIRD includes an abundance of RWD for a large population dispersed across the US. These data have supported many RWE studies completed exclusively within the HIRD [26, 27, 28] and have made the HIRD a valuable contributor to multi‐database efforts [29, 30, 31], including the FDA's Sentinel Initiative [32, 33], Biologics Effectiveness and Safety (BEST) System [34, 35], Innovation in Medical Evidence and Development Surveillance (IMEDS) program [36], Biologics and Biosimilars Collective Intelligence Consortium (BBCIC) [37, 38], and National Evaluation System for Health Technology (NEST) [39, 40]. Carelon Research's unique relationship with Elevance Health enables auditing and investigation of the source of healthcare claims data in the HIRD, providing assurance of data quality and yielding insights that inform study design and interpretation. The data in the HIRD begin in 2006 and are updated monthly, allowing the HIRD to support both historical and ongoing inquiries. The 2020 HIRD population (commercial and managed Medicare) is similar to the 2020 US Census population in terms of sex (male/female; overlap index = 99.2%), age (5‐year age categories; overlap index = 92.0%), and geographic region of residence (Northeast/Midwest/South/West; overlap index = 94.8%). For race/ethnicity (Hispanic or Latino/non‐Hispanic White/non‐Hispanic Black or African American/non‐Hispanic Asian/Other), the overlap index is 86.8%, with the 2020 HIRD population having 13% more non‐Hispanic White members and 11% fewer Hispanic or Latino and non‐Hispanic Black or African American members than the 2020 US Census population [41]. The size of the database, the available data elements, and longitudinal nature of the data allow for a wide variety of pharmacoepidemiologic and health economic and outcomes research studies. The ability to re‐identify individuals (with appropriate approvals) supports many research initiatives, including validation studies [42, 43, 44], patient and provider surveys [45, 46, 47], linkage to medical records [26, 33, 42], linkage to product and disease registries [25, 40, 43, 48], linkage to the NDI [17, 18, 29], as well as recruitment into pragmatic clinical trials [49, 50, 51]. The ability to link family individuals supports pregnancy‐related research [8, 31].

The HIRD also has limitations. Claims data in the HIRD are only captured during periods of enrollment in the health plan, which limits analyses to the time an individual is enrolled in a health plan. Of note, a prior study evaluating enrollment time showed individuals in the HIRD with chronic diseases are enrolled for longer periods of time, allowing for longer follow‐up of this clinically important population [52]. Claims data in the HIRD may incompletely or inaccurately reflect a patient's experience. For example, a pharmacy claim for a prescription medication does not guarantee consumption of the medication, or a medical claim with a diagnosis may be inaccurate due to misdiagnosis or coding errors. Certain data useful for research may not be readily available in the HIRD, such as sample medications from providers, individuals who pay for medications or other services without submitting to the health plan for payment, medications received in inpatient settings, markers of disease severity, or lifestyle risk factors. Limitations of both inaccurate and/or missing data may be addressed through a variety of methods, including the addition of data via linkage to external sources [20, 53], the collection of data directly from patient or provider surveys, and analytically through quantitative bias analysis and other types of sensitivity analyses.

## 6 Data Access

Data within the HIRD are directly available for research through licensing and collaborations with Carelon Research.

## 7 Conclusion

The HIRD, established in 2006, has progressively expanded by incorporating additional data sources, allowing researchers to address more complex questions. This database allows for direct traceability back to the source data, which enhances research integrity and reliability. The HIRD's utility is evidenced by over 1900 peer‐reviewed publications and presentations. The ability to link the HIRD to other datasets amplifies research capabilities and underscores the value of RWD. The HIRD exemplifies how comprehensive data curation can be leveraged for diverse research applications and significant scientific contributions.

### 7.1 Plain Language Summary

This article describes the HIRD, a RWD source that can be used to support health‐related research. The HIRD includes information on individuals with health insurance provided by Elevance Health. These individuals are located throughout the US. The HIRD is built upon the foundation of health insurance enrollment and claims data, which are linked with EHRs, laboratory test results, mortality data, clinical cancer data, social and health equity data, and vaccination data. The data in the HIRD date back to January 2006, and are updated monthly. As of July 2024, the HIRD included over 91 million individuals with medical insurance, including approximately 24 million individuals who were actively enrolled. A strength of the HIRD is that its data can be traced back to their origins. Also, data in the HIRD can be linked to external data sources, and family members within health plans are linked to each other. Because of these and other attributes, the HIRD has been used to support many health‐related research activities over the past 2 decades. The HIRD is directly available for research through licensing and collaborations with Carelon Research.

## Ethics Statement

Carelon Research's access, use, and disclosure of protected health information (PHI) complies with the HIPAA Privacy Rule (45 CFR Part 160 and Subparts A and E of Part 164). Carelon Research does not access, use, or disclose PHI other than as permitted by HIPAA. De‐identified or limited datasets for research are created when feasible; however, when that is not feasible, Carelon Research may seek to obtain a specific waiver of the HIPAA authorization requirements from an Institutional Review Board (IRB). Carelon Research also takes into consideration other federal and state laws and regulations that might limit the use of certain types of data beyond HIPAA limitations, including laws related to substance use disorders and other sensitive medical information.

## Conflicts of Interest

All authors were employees of Carelon Research at the time the manuscript was prepared. All authors are shareholders of Elevance Health. Brett Doherty is currently an employee of Daiichi Sankyo Inc., Basking Ridge, NJ.
